# Unnecessary use of fluoroquinolone antibiotics in hospitalized patients

**DOI:** 10.1186/1471-2334-11-187

**Published:** 2011-07-05

**Authors:** Nicole L Werner, Michelle T Hecker, Ajay K Sethi, Curtis J Donskey

**Affiliations:** 1 School of Medicine, Case Western Reserve University, 10,000 Euclid Avenue, Cleveland, Ohio, USA; 2 Division of Infectious Diseases, MetroHealth Medical Center, 400 MetroHealth Drive, Cleveland, Ohio, USA; 3 Department of Population Health Sciences, University of Wisconsin-Madison School of Medicine and Public Health, Madison, WI, USA; 4 Geriatric Research, Education and Clinical Center, Cleveland Department of Veterans Affairs Medical Center, 10701 East Boulevard, Cleveland, Ohio, USA

## Abstract

**Background:**

Fluoroquinolones are among the most commonly prescribed antimicrobials and are an important risk factor for colonization and infection with fluoroquinolone-resistant gram-negative bacilli and for *Clostridium difficile *infection (CDI). In this study, our aim was to determine current patterns of inappropriate fluoroquinolone prescribing among hospitalized patients, and to test the hypothesis that longer than necessary treatment durations account for a significant proportion of unnecessary fluoroquinolone use.

**Methods:**

We conducted a 6-week prospective, observational study to determine the frequency of, reasons for, and adverse effects associated with unnecessary fluoroquinolone use in a tertiary-care academic medical center. For randomly-selected adult inpatients receiving fluoroquinolones, therapy was determined to be necessary or unnecessary based on published guidelines or standard principles of infectious diseases. Adverse effects were determined based on chart review 6 weeks after completion of therapy.

**Results:**

Of 1,773 days of fluoroquinolone therapy, 690 (39%) were deemed unnecessary. The most common reasons for unnecessary therapy included administration of antimicrobials for non-infectious or non-bacterial syndromes (292 days-of-therapy) and administration of antimicrobials for longer than necessary durations (234 days-of-therapy). The most common syndrome associated with unnecessary therapy was urinary tract infection or asymptomatic bacteriuria (30% of all unnecessary days-of-therapy). Twenty-seven percent (60/227) of regimens were associated with adverse effects possibly attributable to therapy, including gastrointestinal adverse effects (14% of regimens), colonization by resistant pathogens (8% of regimens), and CDI (4% of regimens).

**Conclusions:**

In our institution, 39% of all days of fluoroquinolone therapy were unnecessary. Interventions that focus on improving adherence with current guidelines for duration of antimicrobial therapy and for management of urinary syndromes could significantly reduce overuse of fluoroquinolones.

## Background

Fluoroquinolones are among the most commonly prescribed antibiotics in outpatient and inpatient settings in the United States [1]. Increases in the use of fluoroquinolones in recent years have coincided with steady increases in the incidence of fluoroquinolone-resistance among gram-negative bacilli in intensive care units [2]. In addition, fluoroquinolone exposure has been associated with colonization and infection with other healthcare-associated pathogens, including methicillin-resistant *Staphylococcus aureus *(MRSA), vancomycin-resistant enterococci (VRE), and *Clostridium difficile *[3-5]. Reducing the use of fluoroquinolones may be an effective strategy to limit the dissemination of these pathogens [6,7]. For example, a restriction program that resulted in a 66% reduction in use of fluoroquinolones was associated with control of an outbreak of *C. difficile *infection (CDI) associated with fluoroquinolone-resistant North American pulsed-field gel electrophoresis type 1 (NAP1) strains [7].

Several studies have demonstrated that fluoroquinolones are often used inappropriately [8-12]. Lautenbach et al. [8] found that 81% of fluoroquinolone prescriptions in two academic emergency departments were inappropriate based on institutional guidelines. In a French teaching hospital, Mean et al. [9] found that 51% of fluoroquinolone regimens were innappropriate based on local prescription guidelines. In a tertiary care hospital in Cleveland, we found that 30% of all days of antimicrobial therapy were unnecessary, with ciprofloxacin being the agent most often prescribed unnecessarily [11]. Other small studies have reported frequent misuse of ciprofloxacin and levofloxacin in U.S. hospitals [11,12]. In order to develop effective stewardship interventions, there is a need for additional data on current patterns of inappropriate fluoroquinolone prescribing among hospitalized patients. Therefore, we performed a prospective study to determine the frequency of, reasons for, and adverse effects of unnecessary fluoroquinolone use in a tertiary care medical center. We hypothesized that longer than necessary treatment durations would account for a significant proportion of unnecessary fluoroquinolone use.

## Methods

### Setting

MetroHealth Medical Center is a 650-bed tertiary care hospital in Cleveland, Ohio. The hospital has training programs for residents in internal medicine, family practice, obstetrics and gynecology, and several surgical subspecialties. The fluoroquinolones on formulary are ciprofloxacin and moxifloxacin. Fluoroquinolones are routinely prescribed for prophylaxis prior to invasive urologic procedures, but not for prophylaxis of other procedures or conditions. The hospital does not have a formal antimicrobial stewardship program and there are no restrictions or specific guidelines on fluoroquinolone use. However, pharmacists are assigned to some hospital wards to make antimicrobial recommendations regarding appropriate dosing, potential medication interactions, and potential allergic reactions. Pharmaceutical company representative are allowed to sponsor educational programs for trainees in the hospital and provide promotional materials, but are not allowed to give presentations related to their products. During the year of the study, the percent susceptibilities of *Escherichia coli, Pseudomonas aeruginosa*, and *Klebsiella pneumoniae *to ciprofloxacin were 84%, 75%, and 95%, respectively. The percent susceptibility of *E. coli *to trimethoprim-sulfamethoxazole was 81%.

### Study Design

We prospectively examined the necessity of oral and parenteral fluoroquinolones administered to adult inpatients during a 6-week period in May and June 2009. The study design was based on a previous study of unnecessary use of antimicrobials in our facility [11]. Patients receiving new prescriptions for fluoroquinolones were identified through daily review of pharmacy records. If 10 or less patients received new prescriptions for fluoroquinolones on a given day, all patients were enrolled. If more than 10 patients received new prescriptions, 10 patients were randomly selected to be included in the study using a random number generator. Patients were allowed to be enrolled more than once if they received a second fluoroquinolone regimen at least 4 weeks after completion of the initial regimen. The study wards included 6 medical wards, 3 surgical wards, 3 intensive care units, 1 rehabilitation ward, 1 subacute skilled nursing ward, 1 psychiatric ward, 1 obstetric/gynecologic ward, and the emergency department. Information regarding demographics, admitting service and ward, indication for antimicrobial therapy (prophylaxis versus treatment), concurrent antimicrobials, clinical syndrome being treated, laboratory data, vital signs, radiological tests, and complications of therapy was obtained through medical record review and recorded on a standardized data collection form. Patients were followed through their entire hospital course, including transfers between hospital units.

Patients' medical records were reviewed at least once during the course of antimicrobial therapy and again six weeks after completion of therapy to assess whether possible complications or adverse effects of unnecessary therapy occurred. The frequency of adverse effects was calculated for all unnecessary fluoroquinolone regimens and for those unnecessary regimens in which fluoroquinolones were administered as monotherapy. Colonization or infection with a resistant pathogen (i.e., fluoroquinolone-resistant gram-negative bacillus, vancomycin-resistant enterococci [VRE], or methicillin-resistant *Staphylococcus aureus *[MRSA]) was considered a possible complication of therapy only if the patient did not have a history of colonization or infection with these organisms prior to the start of fluoroquinolone therapy; no routine surveillance for VRE or MRSA was conducted during the study period.

Infectious diseases specialists (M.T.H. and C.J.D.) determined whether the fluoroquinolone regimens were necessary or unnecessary. A fluoroquinolone regimen was defined as unnecessary if no antimicrobial therapy was indicated for the condition being treated or if the fluoroquinolone component of a regimen was not indicated. If the fluoroquinolone was determined to be necessary, additional assessments were made regarding whether part of the fluoroquinolone regimen was unnecessary. Part of the fluoroquinolone regimen was considered unnecessary if the duration of therapy was longer than recommended, the fluoroquinolone provided redundant antimicrobial coverage in the absence of an indication for combination therapy, the fluoroquinolone provided inadequate coverage of expected or documented pathogens, and if the fluoroquinolone was continued despite negative evaluation for infectious syndromes and/or a noninfectious condition was demonstrated to be responsible for the clinical syndrome.

The determination of the necessity of the prescribed fluoroquinolones was based on standard practice guidelines for management of infectious diseases developed and/or endorsed by the Infectious Diseases Society of America [13]. For example, current guidelines for asymptomatic bacteriuria recommend treatment only for pregnant women or individuals undergoing invasive urologic procedures [14]; to diagnose asymptomatic bacteriuria we required documentation that urinary symptoms were not present. If standard practice guidelines were not available, diagnostic and treatment recommendations from a current textbook of infectious diseases were used [14]. Hospital-acquired infections were defined by Centers for Disease Control and Prevention criteria [15]. If fluoroquinolones were considered necessary, we determined if there was an equally effective alternative agent that could have been prescribed. The rationale was to provide an estimate of how much fluoroquinolone use could be safely reduced if a hospital chose to implement a program of complete restriction of fluoroquinolones in an effort to control *C. difficile *or resistant gram-negative bacilli.

To determine the contribution of fluoroquinolones to total antibiotic use in the hospital, we examined pharmacy records for all antibiotics prescribed during the study period. The proportion of total antibiotic use (days of therapy) that was made up of fluoroquinolone use was calculated. In addition, for the year of the study, we estimated the costs of all fluoroquinolone therapy and of the unnecessary fluoroquinolone therapy based on average wholesale prices and on acquisition costs for the hospital.

### Statistical analysis

Data were analyzed with the use of SPSS statistical software version 10.0 (SPSS Inc., Chicago, IL) and STATA 9.1 (StataCorp, College Station, TX). Bivariate analyses were performed to compare necessary and unnecessary treatment regimens. Continuous data were analyzed using student's unpaired *t*-tests. Categorical data were assessed using Pearson Chi-square test or Fisher's exact test. The hospital's institutional review board approved the study protocol and waived the requirement for informed consent due to the observational nature of the study.

## Results

**Figure 1:**
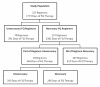
**Overview of Findings Regarding Necessity of Fluoroquinolone Regimens**.

Two hundred twenty-six study subjects received 227 fluoroquinolone regimens during the study period (1 patient was prescribed 2 fluoroquinolone regimens). Figure 1 summarizes the findings regarding the necessity of fluoroquinolone regimens. Of the 227 fluoroquinolone regimens, 70 (31%) were deemed unnecessary. These unnecessary regimens resulted in 391 days of fluoroquinolone therapy, which accounted for 22% of the total days of fluoroquinolone therapy. Analysis of the 157 necessary fluoroquinolone regimens revealed that an additional 299 days of therapy were unnecessary. Overall, 690 of the 1773 (39%) days of fluoroquinolone therapy were deemed unnecessary and 120 of the 226 (53%) study patients received at least one day of unnecessary fluoroquinolone therapy.

Table 1 provides a comparison of the characteristics of patients receiving necessary versus unnecessary fluoroquinolone regimens. Patients receiving unnecessary fluoroquinolone regimens had an older median age than patients receiving necessary regimens (*P *= 0.002); however, there were no significant differences in regards to sex, previous hospitalizations, previous antibiotics, long-term care residence, or comorbidities. A majority of the fluoroquinolone regimens were prescribed on the medical wards or in the emergency department. Only the family practice and rehabilitation medicine wards prescribed significantly more unnecessary than necessary regimens (*P *≤ 0.028).

**Table 1:** Characteristics of 226 Patients Receiving Fluoroquinolone (FQ) Antibiotics

Characteristic	Unnecessary (n = 70)	Necessary (n = 157)	*P *Value
Age in years-median (IQR)	65 (49, 80)	59 (45, 72)	0.045
Males	30 (43)	70 (45)	0.81
Hospitalized in previous 12 months	48 (69)	101 (64)	0.58
Antibiotics in previous 3 months	29 (41)	83 (53)	0.11
FQ antibiotics in previous 3 months	11 (16)	33 (21)	0.35
Long-term care resident	20 (29)	36 (23)	0.38
Comorbidities			
Diabetes	23 (33)	53 (34)	0.89
Cancer	9 (13)	30 (19)	0.25
Chronic neurological condition	17 (24)	28 (18)	0.26
Dementia	12 (17)	16 (10)	0.14
Service First Prescribing FQs			
Emergency Department	22 (31)	55 (35)	0.60
Medicine	25 (36)	68 (43)	0.28
Surgery	6 (9)	21 (13)	0.38
Family Practice	8 (11)	6 (4)	0.04
Rehabilitation Medicine	9 (13)	3 (2)	0.002
Obstetrics/Gynecology	0 (0)	4 (3)	0.31
Indication			
Treatment	68 (97)	150 (96)	0.73
Prophylaxis	2 (3)	7 (4)	
Regimen			
FQ as monotherapy	46 (66)	51 (32)	< 0.001
FQ in combination with other antimicrobials	24 (34)	106 (68)	
Syndrome			
Urinary	36 (51)	28 (18)	< 0.001
Pulmonary	2 (3)	50 (32)	< 0.001
Upper respiratory tract	4 (6)	9 (6)	1.000
Bacteremia	3 (4)	7 (4)	1.000
Fever or sepsis syndrome	3 (4)	11 (7)	0.56
Diarrhea	2 (3)	3 (2)	0.65
Intra-abdominal	2 (3)	20 (13)	0.03
Skin and soft tissue	4 (6)	7 (5)	0.74
Non-infectious condition	11 (16)	18 (11)	0.39
Other	3 (4)	4 (3)	0.68

*Interquartile range (IQR). Data are number of regimens (percentage of regimen category) unless otherwise indicated. Categorical data were assessed using Pearson Chi-square test or Fisher's exact test; Fisher's exact test was used for categorical data when the expected value for any given cell was less than 10*.

Table 2 summarizes the reasons for the unnecessary days of fluoroquinolone therapy. The most common reason for entirely unnecessary fluoroquinolone regimens was treatment of non-infectious or non-bacterial syndromes or of colonization or contamination (292 days of therapy), with asymptomatic bacteriuria accounting for 158 of the unnecessary days. For the necessary regimens, the most common reason for unnecessary days of therapy was longer than necessary treatment (234 days of therapy); longer than necessary therapy was attributable to treatment for longer than recommended durations (121 days of therapy) or failure to discontinue treatment when there was no evidence of infection (113 days of therapy).

**Table 2:** Reasons for Unnecessary Days of fluoroquinolone therapy

	Number of Regimens (Days of Therapy)
Unnecessary fluoroquinolone regimens (n = 70)	
Non-infectious or non-bacterial syndrome	54 (292)
Redundant antimicrobial coverage	10 (58)
Coverage broader than necessary	5 (35)
Inadequate coverage	1 (6)
Part of necessary fluoroquinolone regimen unnecessary (n = 50)	
Duration of treatment longer than necessary	23 (121)
Treatment not discontinued when no evidence of infection	16 (113)
Redundant coverage	4 (21)
Inadequate coverage	7 (44)

Urinary syndromes, including asymptomatic bacteriuria, urinary tract infection, and pyelonephritis, accounted for 51% of the unnecessary fluoroquinolone regimens. In addition, urinary syndromes accounted for 36 of the 299 (12%) days of unnecessary therapy that were prescribed as part of necessary regimens. In total, urinary syndromes accounted for 210 of the 690 (30%) unnecessary days of fluoroquinolone therapy.

For necessary fluoroquinolone regimens, we also assessed whether equally effective alternative agents could have been used for therapy. Of 157 necessary regimens, 87 (55%) could have been replaced with an alternative agent; these regimens accounted for 648 of 1773 (37%) total days of fluoroquinolone therapy. The most common indications for which alternative agents could have been used were gastrointestinal infections (22 regimens, 184 days of therapy), pulmonary infections (21 regimens, 159 days of therapy), and urinary tract infections (21 regimens, 132 days of therapy).

Table 3 shows the adverse events associated with the 120 antibiotic regimens that include unnecessary fluoroquinolone therapy (70 unnecessary regimens and 50 necessary regimens that included unnecessary days of therapy). Gastrointestinal side effects were most frequent adverse events, followed by colonization or infection with resistant organisms, *Clostridium difficile *infection, and *Candida *infection.

**Table 3:** Adverse Events Associated with Unnecessary Fluoroquinolone Antimicrobial Therapy

Adverse Event	All Unnecessary Regimens (n = 70)	*Unnecessary monotherapy regimens (n = 45)	Necessary Regimens with Unnecessary Days of Therapy (n = 50)
GI symptoms	8 (11)	4 (9)	9 (18)
Resistant organism colonization or infection	6 (9)	1 (2)	7 (14)
*Clostridium difficile *infection	0	0	3 (6)
Candida infection	1 (1)	1 (2)	5 (10)
Allergy	2 (3)	0	0
Renal complications	1 (1)	1 (2)	0
Phlebitis	1 (1)	0	0
Other	1 (1)	0	0

Data are presented as number of regimens (percent). * = unnecessary monotherapy regimens are the subset of all unnecessary fluoroquinolone regimens that included only a fluoroquinolone. Resistant organism colonization or infection refers to fluoroquinolone-resistant gram-negative bacilli, vancomycin resistant enterococci, or methicillin-resistant Staphylococcus aureus *isolated from a patient with no previous cultures positive for those organisms. Allergy refers to an allergic reaction (e.g., rash) possibly attributable to fluoroquinolone therapy. The single adverse event that was listed in the other category was hematologic adverse effect*.

During the study period, 11,304 total days of antibiotic therapy were administered to inpatients. Fluoroquinolones accounted for 1,959 of the total days of antibiotic therapy (17%). Ciprofloxacin was the most commonly used fluoroquinolone (1,627 days of therapy); moxifloxacin accounted for the remaining 332 days of therapy. Assuming that fluoroquinolone therapy during the study period was representative of usage patterns throughout the year, the estimated total cost of all of fluoroquinolones administered in 2009 was $205,051 ($127,877 for ciprofloxacin and $77,174 for moxifloxacin). Based on actual pharmacy acquisition charges, the total costs of all fluoroquionolones administered in 2009 was $22,281 ($11,144 for ciprofloxacin and $11,137 for moxifloxacin). If a stewardship program had been effective in eliminating the 31% of fluoroquinolone therapy that was considered unnecessary, the estimated cost savings for the hospital based only on pharmacy expenditures during the year of the study would have been ~$63,566 based on average wholesale prices or $6,907 in actual pharmacy acquisition costs.

## Discussion

We found that 31% of fluoroquinolone regimens prescribed in our institution during the study period were unnecessary. These unnecessary regimens accounted for 22% of all days of fluoroquinolone therapy (391 of 1,773 total days of therapy). The most common reason for unnecessary fluoroquinolone regimens was administration of antimicrobials for non-infectious or non-bacterial syndromes. These findings are consistent with previous studies that evaluated appropriateness of fluoroquinolone therapy regimens [8-11]. For example, Lautenbach et al. [8] found that there was no evidence of infection in 33% of the fluoroquinolone prescriptions that were deemed inappropriate in two emergency departments.

A new observation from our study is that nearly half of all unnecessary days of fluoroquinolone therapy (299 of 690 unnecessary days of therapy) occurred when only part of a treatment regimen was unnecessary. The most common reason for part of a regimen being unnecessary was administration of fluoroquinolones for longer than necessary durations, either because treatment duration was longer than is recommended in current guidelines or because therapy was not discontinued when there was no evidence of infection. These results suggest that interventions to reduce overuse of fluoroquinolones should include efforts to ensure that the duration of therapy is appropriate. Such measures may be clinically important because increased duration of fluoroquinolone therapy has been associated with increased risk of CDI [16]. Efforts to reduce the length of antimicrobial regimens have been advocated as a safe and palatable means to limit overuse of antibiotics [17].

Urinary syndromes were the most common reason for unnecessary fluoroquinolone therapy (30% of all unnecessary days of therapy). Treatment of asymptomatic bacteriuria accounted for 51% of all unnecessary fluoroquinolone regimens, and was particularly common in elderly patients. It is likely that we underestimated the actual number of cases of asymptomatic bacteriuria because documentation of urinary symptoms in the medical record was sometimes lacking and we required documentation that urinary symptoms were not present to classify a case as asymptomatic bacteriuria. Current guidelines for management of asymptomatic bacteriuria recommend treatment only for pregnant women or individuals undergoing invasive urologic procedures [18]. This recommendation is based on randomized trials that have demonstrated that treatment of asymptomatic bacteriuria is not beneficial in other patient populations [18-22]. A recent survey in our hospital demonstrated that most physicians were unaware of the guidelines for management of asymptomatic bacteriuria (authors' unpublished data). To address the overuse of fluoroquinolones in our facility, we have initiated an intervention that focuses primarily on improving adherence to current guidelines for management of asymptomatic bacteriuria and other urinary syndromes. The intervention includes education, audit and feedback regarding appropriateness of therapy, and automated electronic reminders regarding appropriate choice and duration of therapy.

In addition to the evaluation of the necessity of fluoroquinolone therapy, we assessed whether equally effective alternative agents could have been used for therapy. Of the necessary fluoroquinolone regimens, we found that 55% could have been replaced with an alternative agent; these regimens accounted for 37% of the total days of fluoroquinolone therapy. Therefore, our findings suggest that a stewardship program that includes both elimination of unnecessary therapy and formulary substitution when effective alternatives are available could eliminate up to 75% of days of fluoroquinolone therapy without adversely affecting care of patients.

Our study has some limitations. First, the study was conducted in a single tertiary care institution. Additional studies are needed in other tertiary care hospitals and in community hospitals. Second, categorization of therapy as necessary or unnecessary was based on review of medical records, and therefore it is possible that some regimens were misclassified due to inadequate documentation of the indications for treatment. However, as noted previously, the reviewers did not classify therapy as unnecessary in cases where inadequate documentation was available. Third, we examined fluoroquinolone use during a single 6-week period in the springtime. It is possible that fluoroquinolone use may be greater during the winter respiratory virus season. Fourth, we did not include a comparison of the appropriateness of prescribing for physicians at different levels of training. Finally, for regimens in which fluoroquinolones were administered in combination with other agents, it is not possible to determine whether adverse effects were attributable to the fluoroquinolones or to the other agents.

## Conclusions

Fluoroquinolones accounted for 17% of all days of antibiotic therapy in our institution, and 39% of all days of fluoroquinolone therapy were unnecessary. The most common reasons for unnecessary therapy were administration of antimicrobials for non-infectious or non-bacterial syndromes and administration of antimicrobials for longer than recommended durations. These results suggest that interventions that focus on improving adherence with current guidelines for antimicrobial use and for duration of therapy could significantly reduce overuse of fluoroquinolones.

## Competing interests

The authors declare that they have no competing interests.

## Authors' contributions

NFW contributed to the study design, performed medical record review, participated in drafting the manuscript, and participated in drafting and editing the manuscript. MTH contributed to the study design and participated in data collection and analysis. SFG contributed to the study design and participated in data collection. AKS contributed to the study design and performed the data analysis. CJD contributed to the study design, supervised the data collection and analysis, and participated in drafting and editing the manuscript. All of the authors read and approved the final manuscript.

## Pre-publication history

The pre-publication history for this paper can be accessed here:

http://www.biomedcentral.com/1471-2334/11/187/prepub
